# Differential patterns of stromelysin-2 (MMP-10) and MT1-MMP (MMP-14) expression in epithelial skin cancers

**DOI:** 10.1054/bjoc.2000.1634

**Published:** 2001-03

**Authors:** E Kerkelä, R Ala-aho, L Jeskanen, J Lohi, R Grénman, V M-Kähäri, U Saarialho-Kere

**Affiliations:** Department of Dermatology, Helsinki University Central Hospital, Helsinki; Departments of Dermatology and; Otorhinolaryngology–Head and Neck Surgery, Turku University CentralHospital; Department of Medical Biochemistry and MediCity Research Laboratory, University of Turku; Turku Centre for Biotechnology, University of Turku and Åbo Akademi University, Turku, Finland; Department of Pathology, Haartman Institute, University of Helsinki, Finland

**Keywords:** laminin-5, stromelysin-1, cytokine

## Abstract

Co-expression of several members of the matrix metalloproteinase (MMP) family is characteristic of human malignant tumours. To investigate the role of stromelysin-2 (MMP-10) in growth and invasion of skin tumours, we studied cutaneous carcinomas with high metastatic capacity (squamous cell carcinomas, SCCs), only locally destructive tumours (basal cell carcinomas, BCCs) and pre-malignant lesions (Bowen's disease and actinic keratosis) using in situ hybridization. Expression of MMP-10 was compared with that of stromelysin-1 (MMP-3) and of MT1-MMP, the expression of which has been shown to correlate with tumour invasiveness. MMP-10 was expressed in 13/21 SSCs and 11/19 BCCs only in epithelial laminin-5 positive cancer cells, while premalignant lesions were entirely negative. MT1-MMP mRNA was detected in 19/21 SCCs both in epithelial cancer cells and stromal fibroblasts and in 14/18 BCCs only in fibroblasts. The level of MMP-10 was upregulated in a cutaneous SCC cell line (UT-SCC-7) by transforming growth factor-α and keratinocyte growth factor, and by interferon-γ in combination with transforming growth factor-β1 and tumour necrosis factor-α both in UT-SCC-7 and HaCaT cells. Our results show that MMP-10 expression does not correlate with the invasive behaviour of tumours as assessed by their histology and MT1-MMP expression, but may be induced by the wound healing and inflammatory matrix remodelling events associated with skin tumours. © 2001 Cancer Research Campaign http://www.bjcancer.com


British Journal of Cancer (2001) **84**(5), 659–669
© 2001 Cancer Research Campaign
doi: 10.1054/ bjoc.2000.1634, available online at http://www.idealibrary.com on IDEAL
http://www.bjcancer.com

### Differential patterns of stromelysin-2 (MMP-10) and MT1-MMP (MMP-14) expression in epithelial skin cancers

**E Kerkelä[1], R Ala-aho[2,5], L Jeskanen, J Lohi[6], R Grénman[3], V M-Kähäri[4,5] and U Saarialho-Kere[1]**

[1]Department of Dermatology, Helsinki University Central Hospital, Helsinki; [2]Departments of Dermatology and [3]Otorhinolaryngology–Head and Neck Surgery, Turku University Central Hospital; [4]Department of Medical Biochemistry and MediCity Research Laboratory, University of Turku; [5]Turku Centre for Biotechnology, University of Turku and Åbo Akademi University, Turku, Finland; [6]Haartman Institute, Department of Pathology, University of Helsinki, Finland

**Summary** Co-expression of several members of the matrix metalloproteinase (MMP) family is characteristic of human malignant tumours. To investigate the role of stromelysin-2 (MMP-10) in growth and invasion of skin tumours, we studied cutaneous carcinomas with high metastatic capacity (squamous cell carcinomas, SCCs), only locally destructive tumours (basal cell carcinomas, BCCs) and pre-malignant lesions (Bowen's disease and actinic keratosis) using in situ hybridization. Expression of MMP-10 was compared with that of stromelysin-1 (MMP-3) and of MT1-MMP, the expression of which has been shown to correlate with tumour invasiveness. MMP-10 was expressed in 13/21 SSCs and 11/19 BCCs only in epithelial laminin-5 positive cancer cells, while premalignant lesions were entirely negative. MT1-MMP mRNA was detected in 19/21 SCCs both in epithelial cancer cells and stromal fibroblasts and in 14/18 BCCs only in fibroblasts. The level of MMP-10 was upregulated in a cutaneous SCC cell line (UT-SCC-7) by transforming growth factor-α and keratinocyte growth factor, and by interferon-γ in combination with transforming growth factor-β1 and tumour necrosis factor-α both in UT-SCC-7 and HaCaT cells. Our results show that MMP-10 expression does not correlate with the invasive behaviour of tumours as assessed by their histology and MT1-MMP expression, but may be induced by the wound healing and inflammatory matrix remodelling events associated with skin tumours. © 2001 Cancer Research Campaign http://www.bjcancer.com

**Keywords**: laminin-5; stromelysin-1; cytokine

Matrix metalloproteinases (MMPs) are a family of enzymes, which take part in the regulation of the balance between synthesis and breakdown of the extracellular matrix (ECM) (see Birkedal-Hansen et al, 1993). Degradation of ECM is required both in normal physiologic processes, as well as in pathologic tissue remodelling occurring, e.g. in chronic wounds and in cancer. MMPs seem to play an important role in different aspects of tumour progression by enhancing tumour-induced angiogenesis and breaking down ECM and basement membranes (BM) to allow tumour growth and metastatic spread (Kähäri and Saarialho-Kere, 1999).

Stromelysins-1 and -2 (MMPs-3 and -10) are structurally closely related proteolytic enzymes that belong to the MMP gene family. They are highly homologous in primary structure and substrate specificity and they play a critical role in the activation of procollagenases-1 (MMP-1), -2 (MMP-8) and -3 (MMP- 13) (Nicholson et al, 1989; Knäuper et al 1996; Nagase, 1998). Stromelysins-1 and -2 degrade in vitro e.g. collagen III and IV, gelatin, casein, aggrecan, elastin, and proteoglycan core proteins (Murphy et al, 1991; Chandler et al, 1997), while the activity of MMP-10 on fibronectin is negligible compared to MMP-3 (Nagase, 1998).

MMP-10 was initially identified in an adenocarcinoma cDNA library (Muller et al, 1988), and since then its expression has been associated with cancer invasion, mostly in tumours of epithelial origin (Muller et al, 1991; Polette et al, 1991). It was later isolated also from a rheumatoid synovial cell cDNA library and shown to be differentially expressed from stromelysin-1 in human foreskin and rheumatoid synovial fibroblasts (Sirum and Brinckerhoff, 1989). By Northern analysis, MMP-10 has been detected in tumour cells at least in squamous cell carcinomas (SCCs) of the head, neck and lung (Muller et al, 1991), and also in hepatocellular carcinoma using immunoblotting (Lichtinghagen et al, 1995). No MMP-10 expression has been found in colon, gastric or breast adenocarcinomas, substantiating the epithelial origin of MMP-10 in vivo (McDonnell et al, 1991; Heppner et al, 1996). The exact role of MMP-10 in tumour progression is not clearly understood. However, MMP-10 has an important role in cutaneous and intestinal wound healing. It is expressed at the wound edge by migrating basal keratinocytes during both human and murine wound repair (Saarialho-Kere et al, 1994; Madlener et al, 1996) and by migrating enterocytes in inflammatory bowel disease (Vaalamo et al, 1998).

Overexpression of several MMPs correlates with the invasive behaviour of tumour cells (Kähäri and Saarialho-Kere, 1999; Werb, 1999). Expression of MMP-3 has been correlated with tumour progression during skin carcinogenesis (Matrisian et al, 1991). Furthermore, overexpression of MMP-3 in transgenic mice

*Received 12 June 2000*
*Revised 3 October 2000*
*Accepted 18 October 2000*

*Correspondence to*: U Saarialho-Kere was found to be sufficient to generate preneoplastic and malignant mammary gland lesions (Sympson et al, 1995). We have previously demonstrated that MMP-3 and -13 are expressed by stromal and cancer cells in cutaneous squamous cell carcinomas (SCC) (Airola et al, 1997). However, MMP-3 was detected only in the sclerosing subtype of basal cell carcinomas (BCC), which are non-metastasizing, slow-growing but locally invasive and destructive skin tumours. There is no data available on MMP-10 in premalignant skin tumours or BCCs.

MMPs are divided into 5 subgroups (collagenases, gelatinases, stromelysins, membrane-type MMPs, and other MMPs) according to their structure and substrate specificity. The first membrane-type MMP (MT1-MMP) was detected on the surface of invasive tumour cells in 1994 (Sato et al, 1994) and so far, altogether 6 distinct MT-MMPs have been found (Okada et al, 1995; Knäuper and Murphy, 1998; Llano et al, 1999; Pei 1999). MT1-MMP is believed to have an essential role in tumour cell invasion due to its ability to activate latent gelatinase A. The expression of MT1-MMP has been detected in tumour cells and adjacent stromal cells in a variety of human cancers, including e.g. head and neck, vulvar, lung, breast, colon, and liver carcinomas (Sato et al, 1994; Okada et al, 1995; Harada et al, 1998; Johansson et al, 1999). Expression of MT1-MMP is not found in normal epithelial cells, but it is induced in transformed (Kadono et al, 1998) and in epithelial carcinoma cells. High level of MT1-MMP expression has been demonstrated to correlate with invasiveness both in vivo and in vitro in several cancer types (Gilles et al, 1996; Pulyaeva et al, 1997; Mori et al, 1997).

Although the expression of stromelysin-2 has been connected with tumour invasion, its expression in human cancers in vivo is poorly characterized. To investigate the role of stromelysin-2 in skin cancers, we studied its expression in premalignant skin disorders (Bowen's disease and actinic keratosis), non-metastasizing BCCs and in SCCs using in situ hybridization and Northern blot analysis. In this study, we show that MMP-10 mRNA is already induced in BCCs, and co-localizes in epithelial cells with laminin-5. There was no essential correlation with MMP-10 and MT1-MMP expression in skin cancers, suggesting that MMP-10 expression may not necessarily be associated with higher invasive capacity but with the presence of inflammation. The fact that MT1-MMP is expressed in SCCs by cancer cells, but in BCCs only by stromal fibroblasts, may contribute to less invasive behaviour of BCCs as compared with that of SCCs.

### MATERIALS AND METHODS

### Tissue samples

Formalin-fixed, paraffin-embedded specimens of SCC and BCC were obtained from the Department of Dermatopathology, University of Helsinki. The diagnoses were confirmed by 2 experienced dermatopathologists. The following histologic specimens were examined: squamous cell carcinomas (SCC): grades I ($n$ = 9), II ($n$ = 7), III ($n$ = 5), basal cell carcinomas (BCC) ($n$ = 19) (histological subtypes: fibrosing ($n$ = 11), keratotic ($n$ = 3), adenoid ($n$ = 5)), actinic keratoses ($n$ = 6), and Bowen's disease ($n$ = 5).

### In situ hybridization

A 175 bp fragment of human stromelysin-2 (corresponding to nucleotides 1568–1743) and a 217 bp fragment of human stromelysin-1 were used to transcribe sense and anti-sense RNA probes as described previously (Saarialho-Kere et al, 1994). A 1240-bp fragment of MT1-MMP cDNA (Lohi et al, 1996) was subcloned to pGEM vector containing an SP6 RNA polymerase recognition element. When linearized with BgI II, an antisense RNA probe could be transcribed in vitro containing 405 bp from 3UTR of the MT1-MMP cDNA. The specificity of the probe was confirmed by sequencing. As a control for nonspecific hybridization, sections were hybridized with $^{35}$S-labelled sense RNA from bovine tropoelastin cDNA. The validity of this probe as a negative control has been confirmed by Northern (Prosser et al, 1989) and by in situ hybridization (Saarialho-Kere et al, 1992).

All sections were pre-treated with 1 $\mu$g ml$^{-1}$ of proteinase K and washed in 0.1 M triethanolamine containing 0.25% acetic acid. Sections were hybridized with $^{35}$S-labelled probes (2.5–3 $\times$ 10$^4$ cpm $\mu$l$^{-1}$ of hybridization buffer) at 50˚C for at least 18 hours in a humidified chamber. Slides were then washed under stringent conditions, including treatment with RNAse A to remove unhybridized probe (Prosser et al, 1989). After 20–40 days of autoradiography, the photographic emulsion was developed, and the slides were stained with haematoxylin and eosin. Samples previously positive for MMP-10 (chronic and acute wounds) were used as positive controls. The slides were analysed independently by two investigators (US-K, LJ).

### Immunohistochemistry

Immunostaining was performed on sections parallel to those used for in situ hybridization by the avidin-biotin-peroxidase complex technique. Diaminobenzidine (DAB) was used as a chromogenic substrate and haematoxylin as counterstain, as described in detail (Saarialho-Kere et al, 1993). Sections were pre-treated with trypsin (10 mg ml$^{-1}$). Antibodies included polyclonal antibodies against the $\chi^2$-chain of laminin-5 (dilution 1:500, a kind gift from Dr Karl Tryggvason, Karolinska Institut, Sweden), monoclonal transforming growth factor-β1 (TGF-β1) (dilution 1:500, MAB1032, Chemicon International, Temecula, CA), monoclonal anti-MMP-2 (dilution 1:200, GE213, Diabor, Oulu, Finland), and anti-human CD-68 (KP-1, dilution 1:300, M814; Dako, Carpinteria, CA) for tissue macrophages. Controls were performed with rabbit pre-immune serum or normal mouse immunoglobulin.

### Cytokines and growth factors

Human recombinant tumour necrosis factor-α (TNF-α) and TGF-β1 were purchased from Sigma Chemical Co (St Louis, MO). Human recombinant interferon-γ (IFN-γ) was obtained from Promega (Madison, WI). TGF-α, fibroblast growth factor-2 (FGF-2, basic FGF) and keratinocyte growth factor (KGF) were from Pepro Tech EC Ltd (Rocky Hill, NJ).

### Cell cultures

UT-SCC-7 cell line was established from metastasis of a cutaneous SCC at the time of operation in the Turku University Central Hospital. Cell lines were cultured in DMEM supplemented with 6 mM glutamine, non-essential amino acids and 10% fetal calf serum (FCS). The SCC cells were examined in subcultures 5 to 10 and were homogeneous by visual inspection. HaCaT cells, an immortalized human epidermal keratinocyte cell line, were kindly provided by Dr Norbert Fusenig (DKFZ, Heidelberg, Germany).

© 2001 Cancer Research Campaign

HaCaT cells were cultured in Dulbecco's modified Eagle's medium (DMEM) containing 10% FCS.

### RNA analysis

Total cellular RNA was isolated from cell cultures using the single step method (Chomczynski and Sacchi, 1987). Northern blot hybridizations were performed as described previously (Johansson et al, 1997a) with cDNAs labelled with [$\alpha$-$^{32}$P]dCTP using random priming. The 271-bp fragment of MMP-10 cDNA utilized for in situ hybridizations was also used for Northern blot hybridizations. In addition, a 1.3 kb rat cDNA for glyceraldehyde-3-phosphate dehydrogenase (GAPDH) (Fort et al, 1985) was used for hybridizations. [$^{32}$P]-cDNA/mRNA hybrids were visualized by autoradiography.

### RESULTS

### Expression of stromelysin-2 in SSC by cancer cells

Stromelysin-2 was expressed in 13/21 SCC samples by epithelial cancer cells (Figure 1A,B,b and 2A,E). It was generally more abundantly expressed in tumours with a lot of inflammation, near CD-68 positive inflammatory cells (Figure 1A,C). The tumours found negative were typically devoid of inflammation or necrosis (data not shown). There was no clear correlation between the number of cells expressing stromelysin-2 and histological aggressiveness of the tumour (Table 1). However, highly differentiated tumours tended to be negative or showed only a weak signal for stromelysin-2 mRNA (Table 1).

Expression of stromelysin-1 was also studied in a subset of SCC samples (Table 1). Both stromal and tumour cells expressed stromelysin-1 mRNA (Figure 2C) and in only 4/18 samples did it co-localize with stromelysin-2 (Figures 2A,C).

### Stromelysin-2 is not as strongly expressed in BCC as stromelysin-1

Stromelysin-2 mRNA was expressed also in BCCs in 11 of 19 samples studied (Table 1). Of the positive tumours, 8 of 11 represented the fibrosing subtype, and in 6 of them mRNA was detected in cancer cells, while in 2 others the expression was connected with the ulceration (Figure 3A,B,G). In general, the expression was not as abundant as in SCCs and was located near necrotic areas at the tumour surface. Stromelysin-1 expression was in most samples more abundant than that of stromelysin-2, and it was more often expressed by stromal cells surrounding tumour islands than by cancer cells (Figure 3D,H,I,i; Table 1). All specimens of Bowen's disease and actinic keratosis were negative for stromelysin-2, the expression of which has not been found in normal intact skin (Saarialho-Kere et al, 1994). No specific signal was detected using the sense control probe (Figure 1D).

### MT1-MMP mRNA partly co-localizes with stromelysin-2 in SCC, but not in BCC

Production of MT1-MMP was examined, firstly, since its expression in BCCs is poorly characterized and, secondly, since the expression of MT1-MMP may correlate with the invasiveness of the tumour samples under investigation (Gilles et al, 1996; Mori et al, 1997). MT1-MMP mRNA was expressed in 19 of 21 SCCs studied and in 11 of them positive signal was detected in cancer cells (Figure 4B,C,c). Otherwise MT1-MMP was abundantly

**Table 1** Results of MMP-10, MMP-3 and MMP-14 in situ hybridization in squamous cell carcinoma (SCC) and basal cell carcinoma (BCC)

| | | Number of samples/signal strength | | |
|---|---|---|---|---|
| Diagnosis | Total | MMP-10 | MMP-3 | MT1-MMP |
| SCC grade I | 9 | 6/–<br>2/+ca<br>1/++ca | 3/–<br>2/+ca,str<br>1/++ca<br>1/++str | 1/–<br>1/+ca<br>5/+str<br>1/++str |
| SCC grade II | 7 | 1/–<br>3/+ca<br>3/++ca | 2/+ca<br>1/+str<br>1/++str | 1/–<br>1/+str<br>2/+ca,str<br>1/++ca<br>2/+++ca,str |
| SCC grade III | 5 | 1/–<br>3/+ca<br>1/++ca | 1/+ca<br>3/+str | 2/+ca,str<br>2/++ca,str<br>1/++str |
| BCC fibrosing | 11 | 3/–<br>6/+ca<br>2/+ulcer | 2/+ca<br>1/+str<br>1/++str<br>1/+++str | 1/+ca<br>5/+str<br>5/++str |
| BCC keratotic | 3 | 1/–<br>2/+ca | | 3/+str |
| BCC adenoid | 5 | 4/–<br>1/+ca | | 4/– |

In situ hybridization signal for MMPs was evaluated using dark-field microscopy. Signal strength was assessed as follows: –, no detectable specific signal; +, specific signal in few cells; ++, specific signal in moderate number of cells; +++, specific signal in high number of cells. Cells expressing mRNA were identified as cancer cells (ca) or stromal cells (str). In some BCC samples, the signal was detected only in the area of ulceration (ulcer), not at all in cancer cells.

© 2001 Cancer Research Campaign 

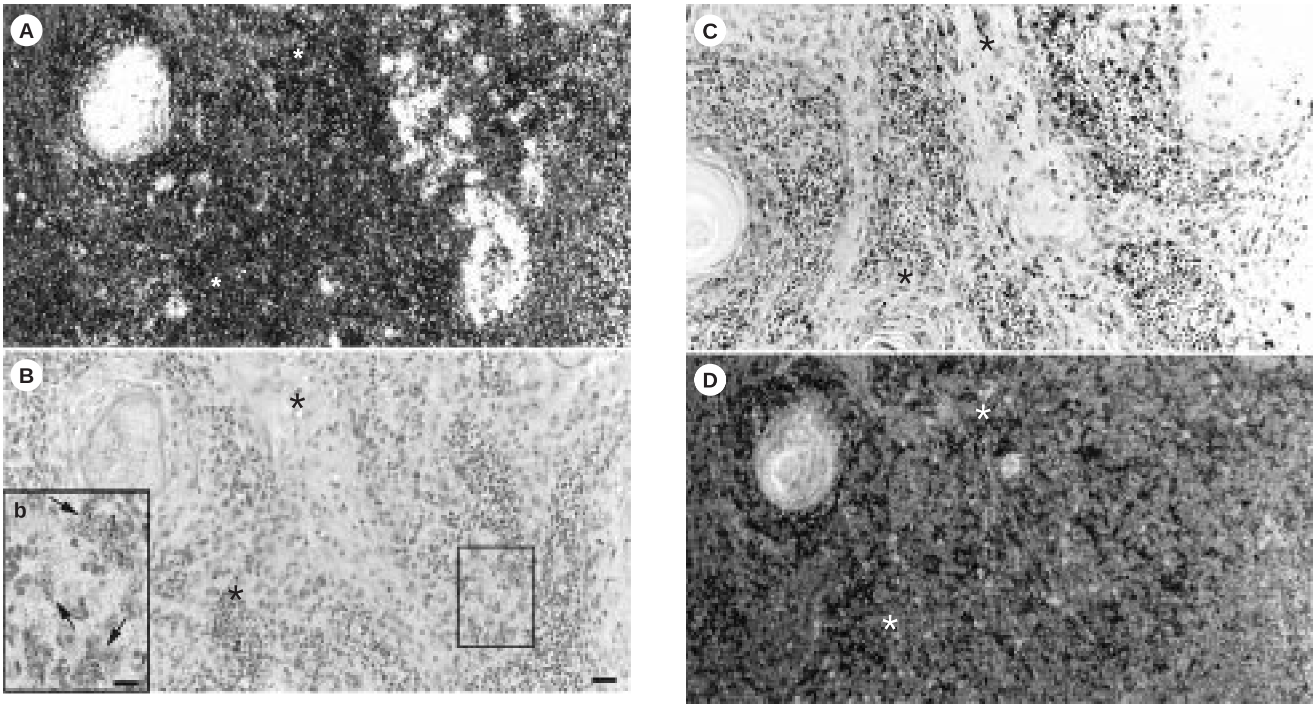

**Figure 1** Expression of MMP-10 in SCCs. (**A**) In situ hybridization dark-field for MMP-10 mRNA in a grade II SCC depicting positive areas inside the tumour. (**B**) A bright-field image of the same tumor. (**b**) A higher magnification of the MMP-10-positive cancer cells (arrows). (**C**) Immunostaining for CD-68 in a nearby section. (**D**) In situ hybridization with the sense probe. Asterisks depict corresponding spots. Counterstaining was performed with haematoxylin and eosin in **A, B, D** and with haematoxylin in **C**. Bars: 50 µm (**A–D**) and 12.5 µm (**b**)

expressed by stromal fibroblasts (Figure 4D,E). There was a partial co-localization of stromelysin-2 and MT1-MMP signal in 4 samples (Figure 4A,B), but usually stromelysin-2 and MT1-MMP had distinct expression patterns. In general, MT1-MMP was expressed deeper at invasive tumour islands (Figure 3A,C).

MT1-MMP mRNA was detected more often than stromelysin-2 mRNA in BCCs. It was expressed in all fibrosing (11) and keratotic (3) BCC samples but not in adenoid BCCs. Unlike in SCCs, cells expressing MT1-MMP were not cancer cells, but stromal fibroblasts surrounding tumour islands (Figures 3C and 4F,G).

### Stromelysin-2 co-localizes with laminin-5 in epithelial cells

Since laminin-5 has been associated with the invasive capacity of several carcinomas (Pyke et al, 1995), immunosignal for laminin-5 was examined in, altogether, 13 SCCs and 11 BCCs. Stromelysin-2-positive cells showed intracellular staining for laminin-5 both in the areas of ulceration as well as at the borders of invasive tumour islands (Figure 2A,B,E,F). However, laminin-5 was clearly more abundantly expressed so that the edges of many tumour islands deeper in the dermis were laminin-5 but not stromelysin-2 positive. Gelatinase A, which is known to be able to degrade laminin-5 in epithelial cells (Giannelli et al, 1999), was not found in the same cells ($n$ = 14 SCCs, $n$ = 6 BCCs), but rather in fibroblasts surrounding cancer islands (data not shown).

Since MT1-MMP has recently been shown to degrade and co-localize with laminin-5 at least in colon and breast cancer (Koshikawa et al, 2000), we also compared its expression to laminin-5 immunopositivity. We found, in altogether 13 SCCs and 11 BCCs, that MT1-MMP mRNA was not expressed in the same cells as laminin-5, but rather in the stromal cells in the vicinity of laminin-5 positive cancer cells (Figure 4D,E).

### Regulation of MMP-10 expression in SCC cells in culture

Previous studies have shown, that the expression of MMP-10 is induced by phorbol ester, TGF-α and epidermal growth factor (EGF) in normal epidermal keratinocytes (Windsor et al, 1993) and by KGF, EGF, TNF-α and TGF-β1 in HaCaT keratinocytes (Madlener et al, 1996). 43 cell lines established from primary and recurrent tumours and metastases of SCCs of the head and neck at the time of operation in the Turku University Central Hospital (Johansson et al, 1997b) were examined for basal expression of MMP-10. No basal MMP-10 mRNA expression was detected in any of the cell lines derived from skin ($n$ = 7), while 2 cell lines from the larynx ($n$ = 12) and 2 from the oral cavity ($n$ = 22) showed some basal expression (Figure 5A and data not shown). To examine the regulation of MMP-10 expression in SCC cells by Northern blot analysis, we chose the UT-SCC-7 a cell line, because it was established from metastasis of a cutaneous SCC. In this context, UT-SCC-7 cells were treated with TGF-α, FGF-2, and KGF, all potent stimulators of keratinocyte proliferation. Marked MMP-10 mRNA abundance was detected in UT-SCC-7 cells treated with TGF-α (10 ng $ml^{-1}$) and KGF (10 ng $ml^{-1}$), whereas FGF-2 (10 ng $ml^{-1}$) had no marked effect (Figure 5A).

We have recently shown, that the expression of MMP-13 and MMP-1 in SCC cells is potently inhibited by IFN-γ (Ala-aho et al, 2000). Treatment of UT-SCC-7 cells with IFN-γ (100 U $ml^{-1}$)

 © 2001 Cancer Research Campaign

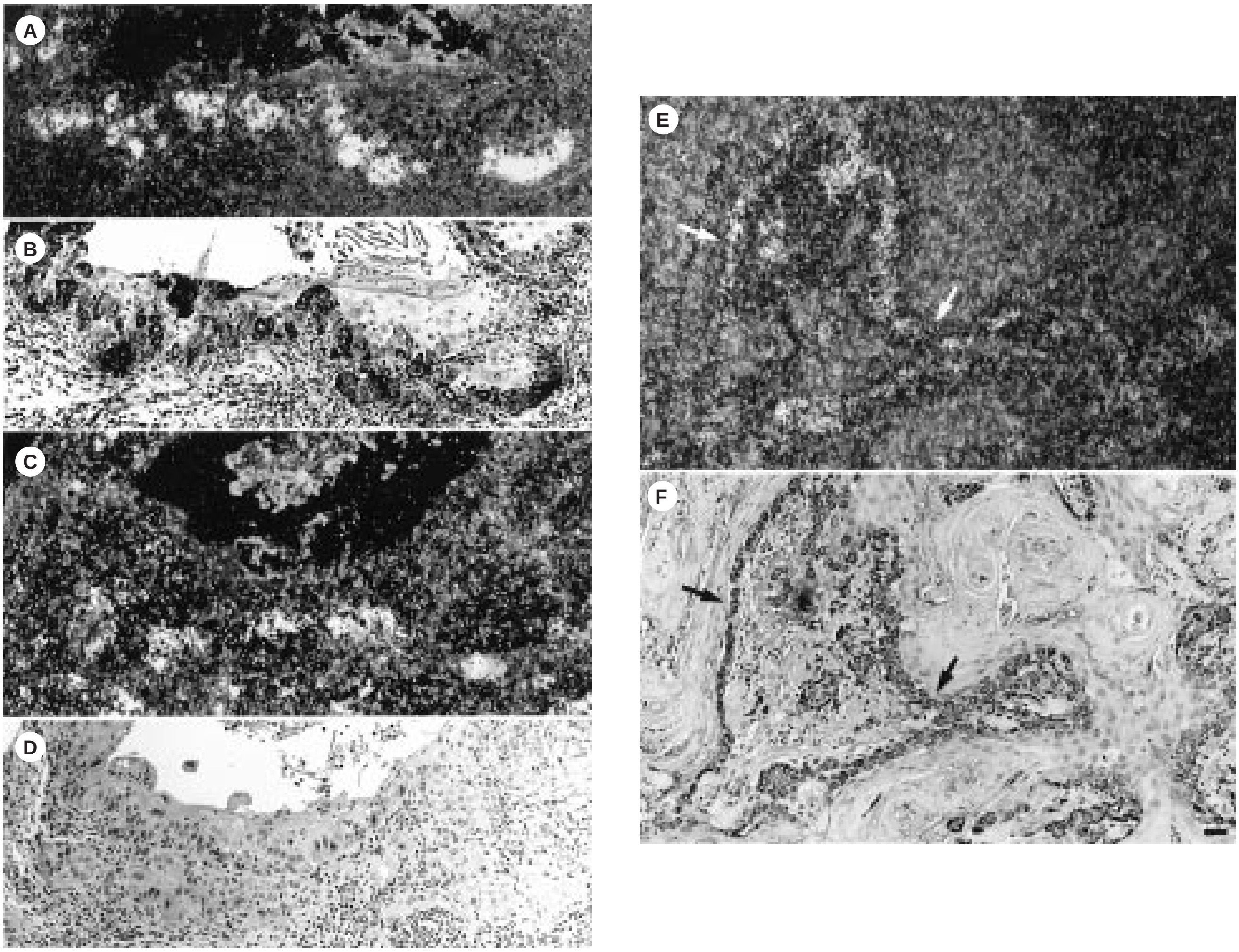

**Figure 2** Expression of MMP-10 and MMP-3 in SCCs. (**A**) In situ hybridization for MMP-10 in a grade I SCC. (**B**) Immunostaining for laminin-5 in a serial section. (**C**) In situ hybridization showing MMP-3 mRNA in a nearby section. (**D**) Negative control for laminin-5 immunostaining. (**E**) Signal for MMP-10 mRNA inside the tumour in a grade III SCC. (**F**) Positive staining for laminin-5 in a serial section. Arrows mark corresponding spots. Counterstaining was performed with haematoxylin and eosin in **A, C, E** and with haematoxylin in **B, D, F**. Bar: 50 μm alone had no effect on MMP-10 mRNA levels, whereas treatment with TGF-β1 (5 ng $ml^{-1}$) and TNF-α (20 ng $ml^{-1}$) slightly enhanced MMP-10 mRNA expression (Figure 5B). Interestingly, exposure of UT-SCC-7 cells to IFN-γ in combination with TGF-β1 or TNF-α resulted in marked induction of MMP-10 expression (Figure 5B). Abundant expression of MMP-10 mRNA was also detected in HaCaT keratinocytes treated with TGF-β1, alone or in combination with IFN-γ (Figure 5C). In contrast to UT-SCC-7 cells, TNF-α alone or in combination with IFN-γ had no marked effect on MMP-10 expression.

Based on our cell culture studies, immunoanalysis for TGF-β1 was performed in cancers. Generally stromelysin-2 positive cells located in the vicinity of TGF-β1-positive areas (Figure 3E,F).

### DISCUSSION

Stromelysin-2 has been thought to contribute to the invasive behaviour of tumour cells. For example, transformed rat embryo cell lines, which had elevated metastatic potential, produced high amounts of both MMP-3 and MMP-10, while their expression was not detected in nonmetastatic cell lines (Sreenath et al, 1992). To date, however, only one report concerning positive MMP-10 expression in human cancers in vivo has been published. Using in situ hybridization, Polette et al (1991) detected MMP-3 and MMP-10 expression in tumour cells arranged along disrupted basement membranes and more often in stromal cells in close contact to cancer cells in head and neck carcinoma. However, their probe was a mixture of stromelysin-1 and -2. Furthermore, upregulation of MMP-10 was detected in human lung and head and neck tumours by Northern analysis; abundant expression of both stromelysin mRNAs, was associated with tumours showing more probable local invasiveness (Muller et al, 1991).

According to our present findings in vivo, despite marked homology of MMP-3 and MMP-10, they still were expressed differently. We found that both of them were upregulated in SCCs and BCCs, but with very distinct patterns. MMP-10 was only expressed by cancer cells in SCCs, which is in contrast with findings on several other MMPs, that are mostly seen in cancer stroma (Werb et al, 1999). The epithelial origin of MMP-10 is further

© 2001 Cancer Research Campaign

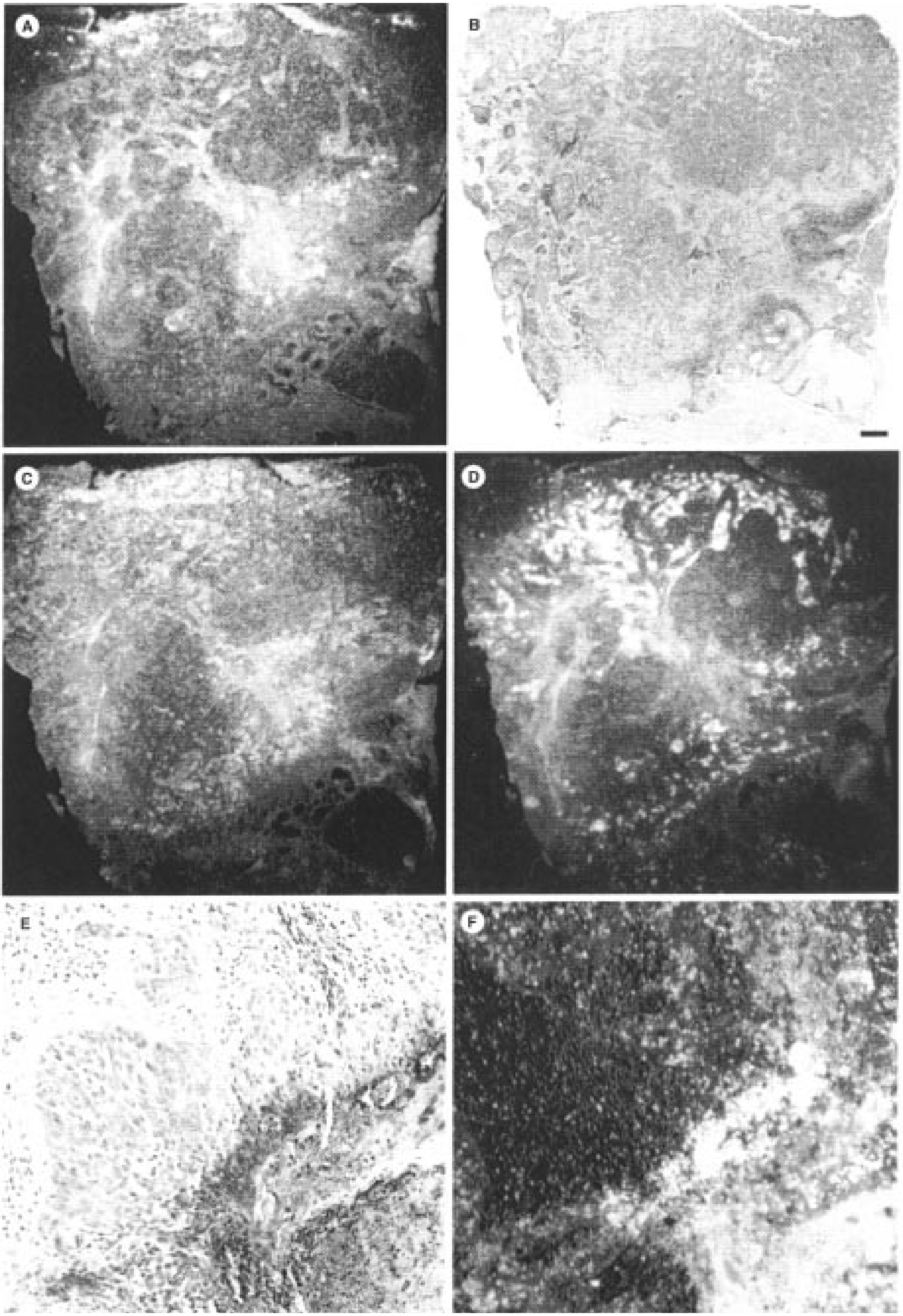

*© 2001 Cancer Research Campaign*

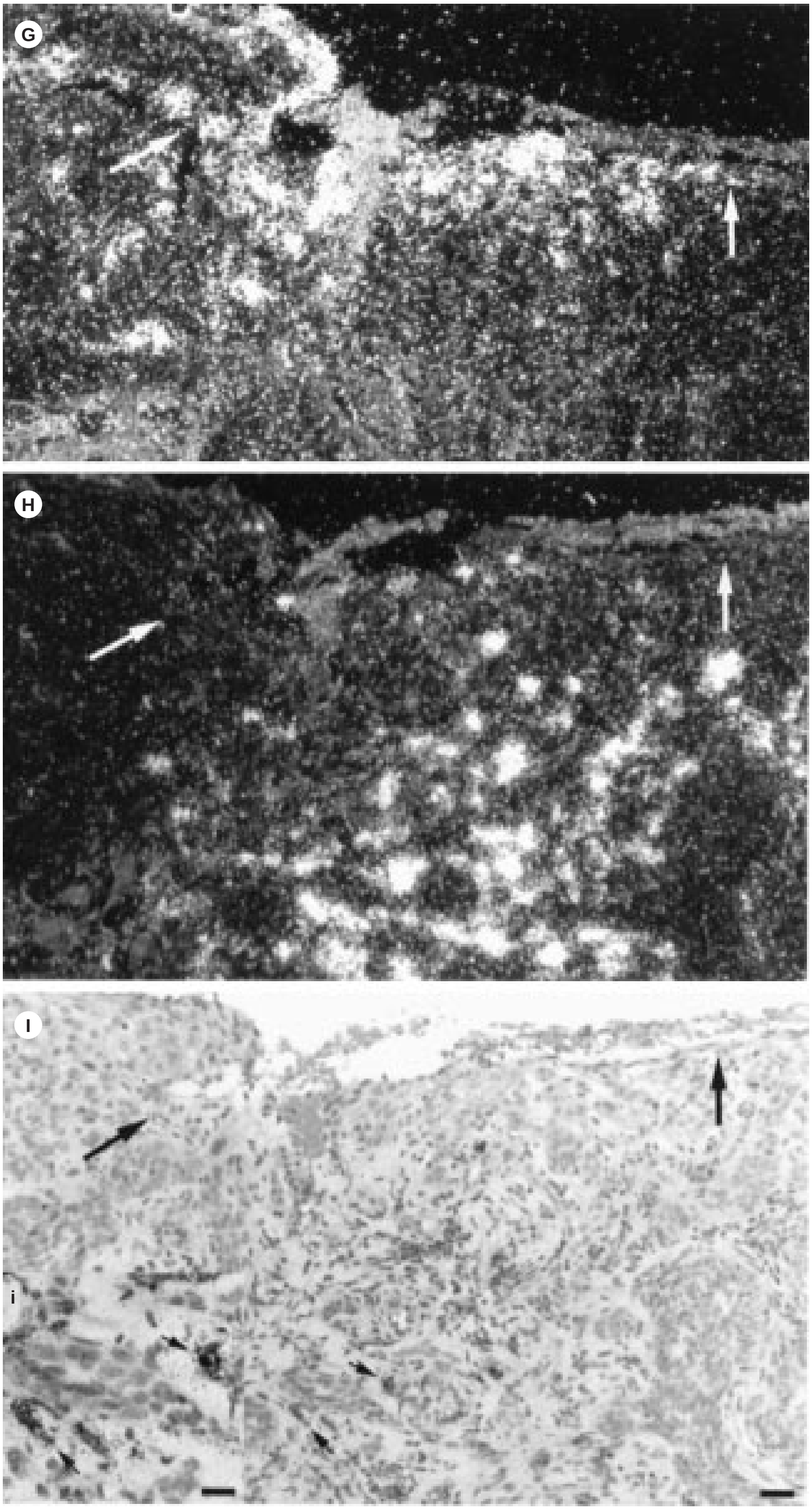

**Figure 3** Expression of MMP-10 and MMP-3 in BCCs. (**A**) In situ hybridization for MMP-10 mRNA in a fibrosing BCC showing expression in cancer cells. (**B**) Corresponding bright-field. (**C**) Signal for MT1-MMP mRNA in a serial section. (**D**) In situ hybridization for MMP-3 also in a serial section. (**E**) Immunostaining for TGF-β1 in another fibrosing BCC. (**F**) MMP-10 mRNA in the same area in a nearby section. (**G**) Higher magnification of the same tumour as in figure A depicting MMP-10 mRNA in epithelial areas. (**H**) Signal for MMP-3 mRNA showing entirely distinct expression pattern. (**I**) Corresponding bright-field image. Arrows mark corresponding spots. (**i**) A closer view of the MMP-3 positive cells in the stroma (small arrows). Counterstaining was performed with haematoxylin and eosin in **A–D, F–I** and with haematoxylin in **E**. Bars: 250 μm (**A–D**), 50 μm (**E–I**), 25 μm (**i**)

*© 2001 Cancer Research Campaign* 

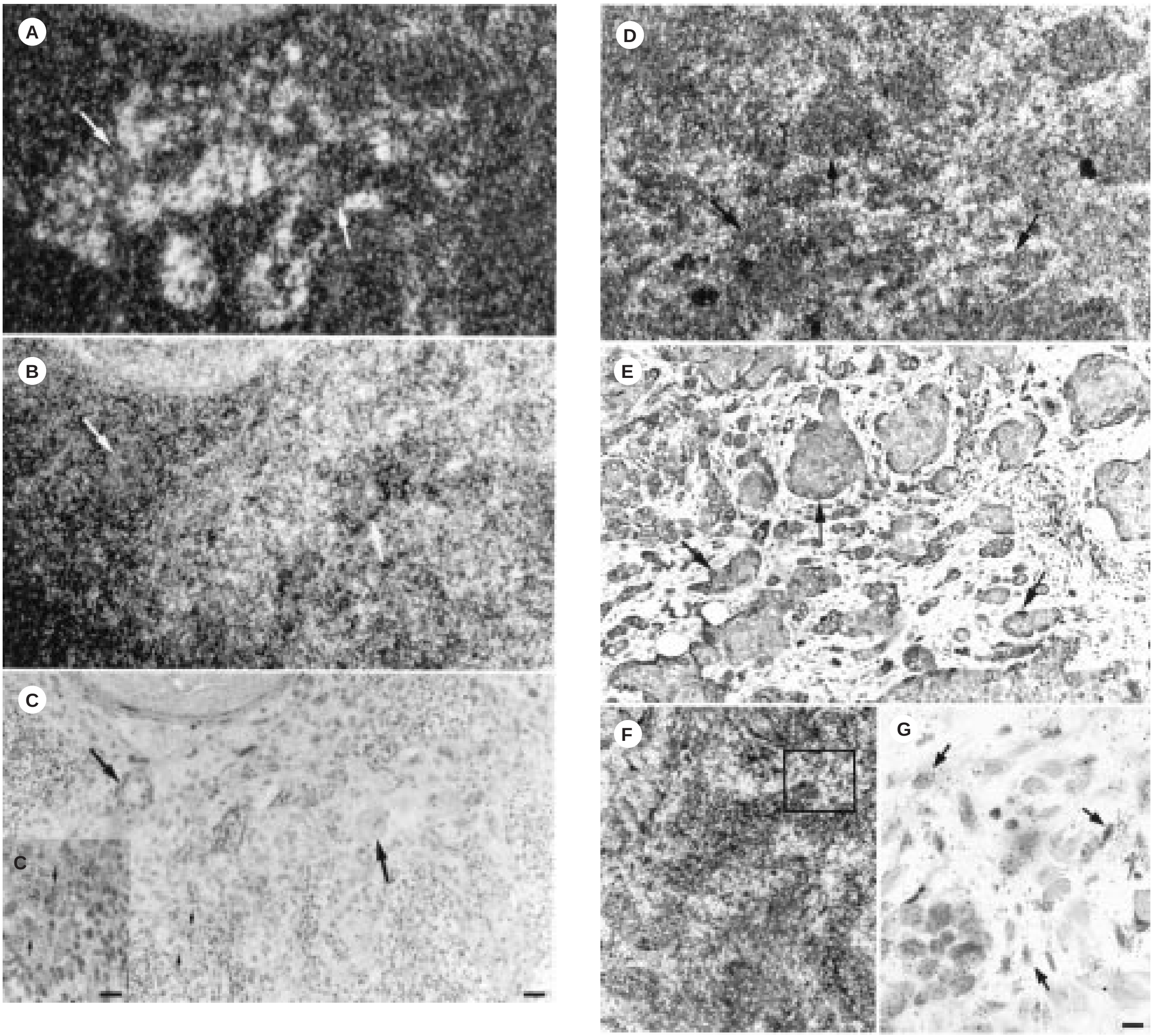

**Figure 4** Expression of MT1-MMP in SCC and BCC. (**A**) A grade II SCC hybridized with MMP-10 antisense probe. (**B**) Signal for MT1-MMP in cancer cells in the same area. (**C**) Corresponding bright-field. Arrows depict corresponding spots. (**C**) A higher magnification of the MT1-MMP positive cells (small arrows). (**D**) Signal for MT1-MMP mRNA in stromal cells in a grade III SCC. (**E**) Immunostaining for laminin-5 in a serial section. Arrows depict corresponding spots. (**F**) A fibrosing BCC island with MT1-MMP mRNA positive cells. (**G**) A closer view of positive fibroblasts (arrows). Counterstaining was performed with haematoxylin and eosin in **A–D, F–G** and with haematoxylin in **E**. Bars: 50 μm (**A–F**), 25 μm (**c**) and 12.5 μm (**G**)

substantiated by our novel results on Barrett's adenocarcinomas in which MMP-10 is only occasionally expressed in epithelial cells in conjunction with ulceration (Salmela and Saarialho-Kere, unpublished data). In BCCs, MMP-10 was also expressed in cancer cells, except when the expression was connected with the ulceration. In general, the expression was more sporadic than in SCCs and located near necrotic areas at the tumour surface. MMP-3 was more often expressed by stromal cells both in SCCs and BCCs and was clearly more abundant than MMP-10 in BCCs (Table 1). No signal for MMP-10 was detected in Bowen's disease or actinic keratoses, in agreement with data on MMP-3 (Tsukifuji et al, 1999). Differential expression patterns of MMP-3 and -10 have also been demonstrated in developing human bone (Bord et al, 1998) and during cutaneous wound repair (Saarialho-Kere et al, 1994). It seems that they both have important, but still unique roles in the degradation of ECM macromolecules in cancer.

MMP-3 is considered to be an essential activator of various proMMPs. Having a similar substrate specificity, also MMP-10 is able to cleave several inactive proMMPs including MMPs-1, -7, -8, -9, and -13 (Karelina et al, 1994; Knäuper et al, 1996b; Nakamura et al, 1998) of which at least MMP-9 and MMP-13 are considered to be particularly important in the malignant behaviour of the tumour cells. In skin cancers, MMP-10 could activate MMP-9 or MMP-7 (Murphy et al, 1991). Pro-MMP-10 might be activated by serine proteases known to be present in skin cancers (see Nagase, 1998).

Laminin-5, the main component of anchoring filaments, plays a major part in keratinocyte adhesion and migration (Yancey, 1995).

© 2001 Cancer Research Campaign

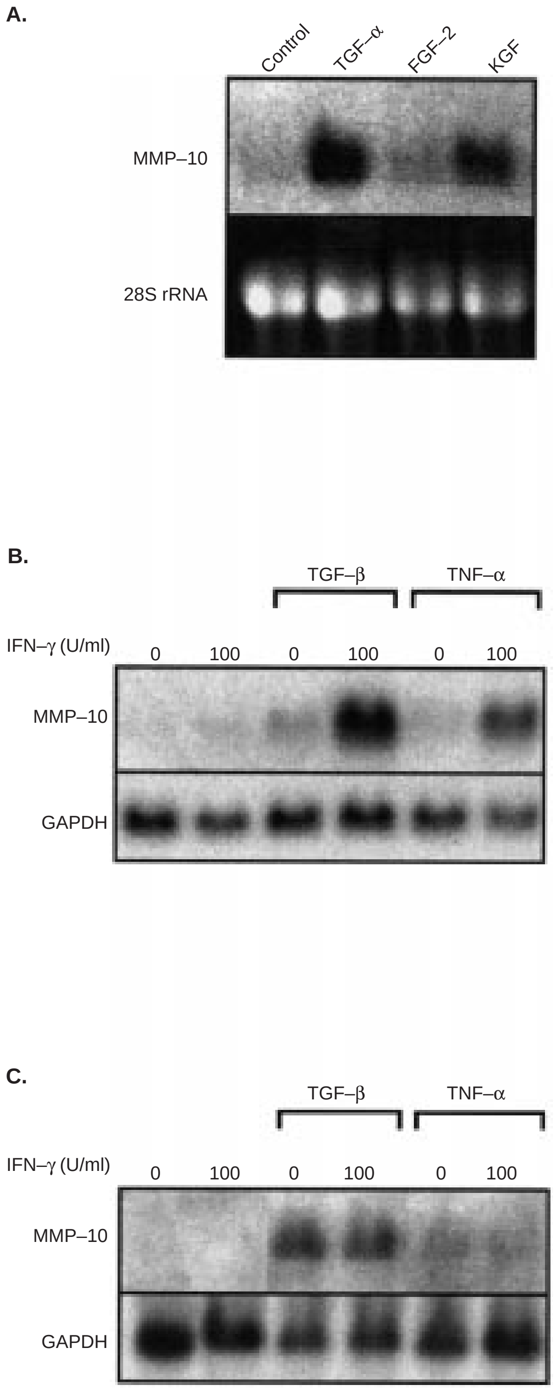

**Figure 5** Enhancement of MMP-10 mRNA levels in SCC cells by TNF-α, TGF-β1, TGF-α, and KGF. Cell line UT-SCC-7 established from a metastasis of cutaneous SCC was incubated for 24 h in serum free medium (control), or in the presence of (**A**) TGF-a (10 ng ml$^{-1}$), FGF-2 (10 ng ml$^{-1}$), or KGF (10 ng ml$^{-1}$). (**B**) UT-SCC-7 cells and (**C**) HaCaT keratinocytes were treated with IFN-g (100 U ml$^{-1}$) for 1 h prior to adding TGF-b1 (5 ng ml$^{-1}$) or TNF-α (20 ng ml$^{-1}$) for 24 h. Aliquots of total RNA (20 mg lane$^{-1}$) were analysed by Northern blot hybridization with cDNA probes specific for MMP-10 and GAPDH. 28 S rRNA was visualized by ethidium bromide staining

Laminin-5 has been suggested to aid in the migration and invasion of tumour cells by establishing focal adhesions to the matrix (Pyke et al, 1995). According to Skyldberg et al (1999) γ-chain of laminin-5 co-localizes with the invasive cells in cervical lesions and, thus, can serve as a marker for early invasion. In the samples examined, MMP-10 mRNA was expressed by epithelial cells producing laminin-5, implicating a migratory status of these cells. However, laminin-5 was expressed more abundantly than MMP-10, particularly deeper in the invasive borders of tumour islands. Gelatinase A activity induces migration of breast epithelial cells by cleaving and regulating the function of laminin-5 (Giannelli et al, 1999) and while this study was in progress, Koshikawa et al (2000) showed that this also applies to MT1-MMP. In our own SCC samples ($n$ = 13), MT1-MMP and laminin-5 did not generally co-localize in the same cells, but MT1-MMP mRNA located in stromal cells next to laminin-5 expressing cancer cells (Figure 4D,E). Thus, we cannot exclude that either MT1-MMP or MMP-10 or both, might cleave laminin-5 during keratinocyte motility in skin cancer. Instead, gelatinase A protein did not co-localize with laminin-5 and apparently does not cleave laminin-5 during skin carcinogenesis. Interestingly, TGF-β1 is able to induce both MMP-10 (Madlener et al, 1996) and laminin-5 in keratinocytes (Kainulainen et al, 1998).

Cultured human foreskin keratinocytes express MMP-10, while fibroblasts express MMP-3. However, same agents, such as phorbol esters, TNF-α, TGF-α, and EGF up-regulate the expression of both stromelysins (Windsor et al, 1993; Nagase, 1998). The expression of MMP-10 is also induced by KGF and TGF-β1 in human transformed keratinocytes (HaCaT) (Madlener et al, 1996), that in some aspects mimic the phenotype of BCCs. Interestingly, most of the SCC cell lines previously shown to be producing MMP-1 and -13 basally (Johansson et al, 1997b), were negative for MMP-10 mRNA. This suggests that malignant transformation per se is not enough to induce MMP-10, but rather inflammatory cytokines modify its expression.

Phorbol ester stimulates the production of MMP-10 in certain SCC cell lines, while IL-1β has no inducible effect (Nakamura et al, 1998). Tumour-infiltrating lymphocytes in SCCs express IFN-γ, which can inhibit proliferation of SCC cells (Kim et al, 1995; Arany et al, 1997) and stimulate production of MMP-1 and MMP-3 by normal epidermal keratinocytes (Tamai et al, 1995). Inflammatory cells may produce factors, eg. TNF-α and TGF-β1, which can stimulate production of invasion-associated proteinases by tumour and stromal cells. In our UT-SCC-7 cell line, TGF-α, KGF and TGF-β1 clearly upregulated MMP-10. IFN-γ in combination with TGF-β1 and TNF-α resulted in marked induction of MMP-10. Interestingly, the expression of 2 other MMPs relevant in cancer biology, MMP-1 and -13 are also upregulated by the same cytokines (Johansson et al, 1997b) but inhibited by IFN-γ (Ala-aho et al, 2000). The clinical picture of SCCs is characterized by ulceration and inflammation of the lesions. Therefore, it is possible that mediators of wound repair and inflammation, such as TGF-β1, IFN-γ, KGF, EGF and TNF-α, modulate invasion of SCCs and thus MMP-10 upregulation can also be detected. In agreement with that hypothesis, a positive immunostaining for TGF-β1 was seen in the same area as MMP-10 mRNA. Furthermore, it has been shown, that high expression of IFN-γ in SCCs correlates with favourable prognosis (Arany et al, 1997). This observation, in the context of our finding that IFN-γ stimulates SCC cell MMP-10 expression, suggests that MMP-10 is not involved in the invasion of SCC cells.

Membrane-associated MMPs (MT-MMPs) seem to have an essential role in pericellular activation cascades of MMPs. MT1-MMP at the cell surface, for example, may trigger invasion of tumour cells by activation of pro-gelatinase A produced by stromal cells (see Knäuper and Murphy, 1998). MT1-MMP mRNA has been

*© 2001 Cancer Research Campaign* 

detected both in cancer and stromal cells (Okada et al, 1995; Heppner et al, 1996; Ohtani et al, 1996; Harada et al, 1998). This correlates with our results, since we found MT1-MMP mRNA expression both in cancer cells and in stromal fibroblasts surrounding SCCs. Ohtani et al (1996) suggested that the expression of MT1-MMP in cancer cells might relate to invasive growth, while in fibroblasts it may be implicated in tissue remodelling processes caused by invasive cancer cells since it is able to digest types I and III collagens, fibronectin, vitronectin, and proteoglycans (d'Ortho et al, 1997; Ohuchi et al, 1997). According to our results, MT1-MMP-positive cancer cells were detected especially at the invasive edge of tumour islands. Interestingly, MT1-MMP can activate latent MMP-13 (Knäuper et al, 1996c) and based on our previous data (Airola et al, 1997), may be serving this function at the epithelial front.

To our knowledge, this is the first report on MT1-MMP expression in BCCs. Unlike in SCCs, MT1-MMP expression was found generally in stromal fibroblasts surrounding tumour islands. This suggests that MT1-MMP produced by fibroblasts in BCC might have another task than that produced by cancer cells in invasive SCCs. The non-invasive nature of BCC supports this result. Yoshizaki et al (1997) connected high levels of MT1-MMP expression to highly differentiated head and neck SCCs. According to our results, we found no significant difference in intensity of MT1-MMP expression between poorly or well-differentiated cutaneous tumours.

In conclusion, this is the first study showing differential expression of stromelysin-1 and -2 in human cancer tissues in vivo. Conversely to some previous in vitro reports, the present study shows that stromelysin-2 expression does not correlate with the invasive behaviour of tumours as assessed by their histology and MT1-MMP expression, but may be induced by several cytokines functioning in ulcerative phenomena and inflammatory matrix remodelling associated with skin tumours.

### ACKNOWLEDGEMENTS

We thank Mrs Alli Tallqvist for skillful technical assistance. Supported by grants from Helsinki and Turku University Central Hospital Research Foundations, the Academy of Finland, the Sigrid Juselius Foundation, Cancer Research Foundation of Finland, and The Finnish Cultural Foundation.

### REFERENCES

Airola K, Johansson N, Kariniemi AL, Kähäri VM and Saarialho-Kere UK (1997) Human collagenase-3 is expressed in malignant squamous epithelium of the skin. *J Invest Dermatol* **109**: 225–231

Ala-aho R, Johansson N, Grénman R, Fusenig NE, López-Otín C and Kähäri V-M (2000) Inhibition of collagenase-3 (MMP-13) expression in transformed human keratinocytes by interferon-γ is associated with activation of extracellular signal-regulated kinase-1,2 and STAT1. *Oncogene* **19**: 248–257

Arany I, Fleischmann CM, Tyring SK and Fleischmann WR Jr (1997) Lack of mda-6/WAF1/CIP1-mediated inhibition of cyclin-dependent kinases in interferon-alpha resistant murine B16 melanoma cells. *Cancer Lett* **119**: 237–240

Birkedal-Hansen H, Moore WG, Bodden MK, Windsor LJ, Birkedal-Hansen B, De Carlo A and Engler JA (1993) Matrix metalloproteinases: a review. *Crit Rev Oral Biol Med* **4**: 197–250

Bord S, Horner A, Hembry RM and Compston JE (1998) Stromelysin-1 (MMP-3) and stromelysin-2 (MMP-10) expression in developing human bone: potential roles in skeletal development. *Bone* **23**: 7–12

Chandler S, Miller KM, Clements JM, Lury J, Corkill D, Anthony DC, Adams SE and Gearing JA (1997) Matrix metalloproteinases, tumor necrosis factor and multiple sclerosis: an overview. *J Neuroimmunol* **72**: 155–161

Chomczynski P and Sacchi N (1987) Single-step method of RNA isolation by acid guanidinium thiocyanate-phenol-chloroform extraction. *Anal Biochem* **162**: 156–159

d'Ortho MP, Will H, Atkinson S, Butler G, Messent A, Gavrilovic J, Smith B, Timpl R, Zardi L and Murphy G (1997) Membrane-type matrix metalloproteinases 1 and 2 exhibit broad-spectrum proteolytic capacities comparable to many matrix metalloproteinases. *Eur J Biochem* **250**: 751–757

Fort P, Marty L, Piechaczyk M, El Sabrouty S, Dani C, Jeanteur P and Blanchard JM (1985) Various rat adult tissues express only one major mRNA species from the glyceraldehyde-3-phosphate-dehydrogenase multigenic family. *Nucleic Acids Res* **13**: 1431–1441

Giannelli G, Pozzi A, Stetler-Stevenson WG, Gardner HA and Quaranta V (1999) Expression of matrix metalloprotease-2-cleaved laminin-5 in breast remodeling stimulated by sex steroids. *Am J Pathol* **154**: 1193–1201

Gilles C, Polette M, Piette J, Munaut C, Thompson EW, Birembaut P and Foidart JM (1996) High level of MT-MMP expression is associated with invasiveness of cervical cancer cells. *Int J Cancer* **65**: 209–213

Harada T, Arii S, Mise M, Imamura T, Higashitsuji H, Furutani M, Niwano M, Ishigami S, Fukumoto M, Seiki M, Sato H and Imamura M (1998) Membrane-type matrix metalloproteinase-1 (MT1-MTP) gene is overexpressed in highly invasive hepatocellular carcinomas. *J Hepatol* **28**: 231–239

Heppner KJ, Lynn MM, Jensen RA and Rodgers WH (1996) Expression of most matrix metalloproteinase family members in breast cancer represents a tumor-induced host response. *Am J Pathol* **149**: 273–282

Johansson N, Westermarck J, Leppä S, Häkkinen L, Koivisto L, López-Otín C, Peltonen J, Heino J and Kähäri VM (1997a) Collagenase-3 (MMP-13) gene expression by HaCaT keratinocytes is enhanced by tumor necrosis factor-α and transforming growth factor-β. *Cell Growth Differ* **8**: 243–250

Johansson N, Airola K, Grenman R, Kariniemi AL, Saarialho-Kere U and Kähäri VM (1997b) Expression of collagenase-3 (matrix metalloproteinase-13) in squamous cell carcinomas of the head and neck. *Am J Pathol* **151**: 499–508

Johansson N, Vaalamo M, Grénman S, Hietanen S, Klemi P, Saarialho-Kere U and Kähäri VM (1999) Collagenase-3 (MMP-13) is expressed by tumor cells in invasive vulvar squamous cell carcinomas. *Am J Pathol* **154**: 469–480

Kadono Y, Okada Y, Namiki M, Seiki M and Sato H (1998) Transformation of epithelial Madin-Darby canine kidney cells with p60(v-src) induces expression of membrane-type 1 matrix metalloproteinase and invasiveness. *Cancer Res* **58**: 2240–2244

Kähäri V-M and Saarialho-Kere U (1999) Matrix metalloproteinases and their inhibitors in tumour growth and invasion. *Ann Med* **31**: 34–45

Kainulainen T, Häkkinen L, Hamidi S, Larjava K, Kallioinen M, Peltonen J, Salo T, Larjava H and Oikarinen A (1998) Laminin-5 expression is independent of the injury and the microenvironment during reepithelialization of wounds. *J Histochem Cytochem* **46**: 353–360

Karelina TV, Goldberg GI and Eisen AZ (1994) Matrilysin (PUMP) correlates with dermal invasion during appendageal development and cutaneous neoplasia. *J Invest Dermatol* **103**: 482–487

Kim J, Modlin RL, Moy RL, Dubinett SM, McHugh T, Nickoloff BJ and Uyemura K (1995) IL-10 production in cutaneous basal and squamous cell carcinomas. A mechanism for evading the local T cell immune response. *J Immunol* **155**: 2240–2247

Knäuper V and Murphy G (1998) Membrane-type matrix metalloproteinases and cell-surface-associated activation cascades for matrix metalloproteinases. In: *Matrix metalloproteinases*. Parks WC and Mecham RP (eds) pp. 199–218. Academic Press, San Diego

Knäuper V, Lopez-Otin C, Smith B, Knight G and Murphy G (1996a) Biochemical characterization of human collagenase-3. *J Biol Chem* **271**: 1544–1550

Knäuper V, Murphy G and Tschesche H (1996b) Activation of human neutrophil procollagenase by stromelysin 2. *Eur J Biochem* **235**: 187–191

Knäuper V, Will H, Lopez-Otin C, Smith B, Atkinson SJ, Stanton H, Hembry RM and Murphy G (1996c) Cellular mechanisms for human procollagenase-3 (MMP-13) activation. Evidence that MT1-MMP (MMP-14) and gelatinase a (MMP-2) are able to generate active enzyme. *J Biol Chem* **271**: 17124–17131

Koshikawa N, Giannelli G, Cirulli V, Miyazaki K and Quaranta V (2000) Role of cell surface metalloprotease MT1-MMP in epithelial cell migration over laminin-5. *J Cell Biol* **148**: 615–624

Lichtinghagen R, Helmbrecht T, Arndt B and Boker KH (1995) Expression pattern of matrix metalloproteinases in human liver. *Eur J Clin Chem Clin Biochem* **33**: 65–71

Llano E, Pendas AM, Freije JP, Nakano A, Knäuper V, Murphy G and Lopez-Otin C (1999) Identification and characterization of human MT5-MMP, a new

© 2001 Cancer Research Campaign membrane-bound activator of progelatinase a overexpressed in brain tumors. *Cancer Res* **59**: 2570–2576

Lohi J, Lehti K, Westermarck J and Kähäri V-M and Keski-Oja J (1996) Regulation of membrane type metalloproteinase-1 expression by growth factors and phorbol 12-myristate 13-acetate. *Eur J Biochem* **239**: 239–247

Madlener M, Mauch C, Conca W, Brauchle M, Parks WC and Werner S (1996) Regulation of the expression of stromelysin-2 by growth factors in keratinocytes: implications for normal and impaired wound healing. *Biochem J* **320**: 659–664

Matrisian LM, McDonnell S, Miller DB, Navre M, Seftor EA and Hendrix MJ (1991) The role of the matrix metalloproteinase stromelysin in the progression of squamous cell carcinomas. Am *J Med Sci* **302**: 157–162

McDonnell S, Navre M, Coffey RJ Jr and Matrisian LM (1991) Expression and localization of the matrix metalloproteinase pump-1 (MMP-7) in human gastric and colon carcinomas. *Mol Carcinog* **4**: 527–533

Mori M, Mimori K, Shiraishi T, Fujie T, Baba K, Kusumoto H, Haraguchi M, Ueo H and Akiyoshi T (1997) Analysis of MT1-MMP and MMP2 expression in human gastric cancers. *Int J Cancer* **74**: 316–321

Muller D, Quantin B, Gesnel MC, Millon-Collard R, Abecassis J and Breathnach R (1988) The collagenase gene family in humans consists of at least four members. *Biochem J* **253**: 187–192

Muller D, Breathnach R, Engelmann A, Millon R, Brenner G, Flesch H, Dumont P, Eber M and Abecassis J (1991) Expression of collagenase-related metalloproteinase genes in human lung or head and neck tumours. *Int J Cancer* **48**: 550–556

Murphy G, Cockett MI, Ward RV and Docherty AJP (1991) Matrix metalloproteinase degradation of elastin, type IV collagen and proteoglycan. A quantitative comparison of the activities of 95 kDa and 72 kDa gelatinases, stromelysins-1 and -2 and punctuated metalloproteinase (PUMP). *Biochem J* **277**: 277–279

Nagase H (1998) Stromelysins 1 and 2. In: *Matrix metalloproteinases*. Parks WC and Mecham RP (eds) pp. 43–84. Academic Press, San Diego

Nakamura H, Fujii Y, Ohuchi E, Yamamoto E and Okada Y (1998) Activation of the precursor of human stromelysin 2 and its interactions with other matrix metalloproteinases. *Eur J Biochem* **253**: 67–75

Nicholson R, Murphy G and Breathnach R (1989) Human and rat malignant-tumor-associated mRNAs encode stromelysin-like metalloproteinases. *Biochemistry* **28**: 5195–5203

Ohtani H, Motohashi H, Sato H, Seiki M and Nagura H (1996) Dual over-expression pattern of membrane-type metalloproteinase-1 in cancer and stromal cells in human gastrointestinal carcinoma revealed by in situ hybridization and immunoelectron microscopy. *Int J Cancer* **68**: 565–570

Ohuchi E, Imai K, Fujii Y, Sato H, Seiki M and Okada Y (1997) Membrane type 1 matrix metalloproteinase digests interstitial collagens and other extracellular matrix macromolecules. *J Biol Chem* **272**: 2446–2451

Okada A, Bellocq JP, Rouyer N, Chenard MP, Rio MC, Chambon P and Basset P (1995) Membrane-type matrix metalloproteinase (MT-MMP) gene is expressed in stromal cells of human colon, breast, and head and neck carcinomas. *Proc Natl Acad Sci* USA **92**: 2730–2734

Pei D (1999) Leukolysin/MMP25/MT6-MMP: a novel matrix metalloproteinase specifically expressed in the leukocyte lineage. *Cell Res* **9**: 291–303

Polette M, Claver C, Muller D, Abecassis J, Binninger I and Birembaut P (1991) Detection of mRNAs encoding collagenase-1 and stromelysin-2 in carcinomas of the head and neck by in situ hybridization. *Invasion Metast* **11**: 76–83

Prosser IW, Stenmark KR, Suthar M, Crouch EC, Mecham RP and Parks WC (1989) Regional heterogeneity of elastin and collagen gene expression in intralobar arteries in response to hypoxic pulmonar hypertension as demonstrated by in situ hybridization. *Am J Pathol* **135**: 1073–1088

Pulyaeva H, Bueno J, Polette M, Birembaut P, Sato H, Seiki M and Thompson EW (1997) MT1-MMP correlates with MMP-2 activation potential seen after epithelial to mesenchymal transition in human breast carcinoma cells. *Clin Exp Metastasis* **15**: 111–120

Pyke C, Salo S, Ralfkiaer E, Romer J, Dano K and Tryggvason K (1995) Laminin-5 is a marker of invading cancer cells in some human carcinomas and is coexpressed with the receptor for urokinase plasminogen activator in budding cancer cells in colon adenocarcinomas. *Cancer Res* **55**: 4132–4139

Saarialho-Kere UK, Chang ES, Welgus HG and Parks WC (1992) Distinct localization of collagenase and TIMP expression in wound healing associated with ulcerative pyogenic granuloma. *J Clin Invest* **90**: 1952–1957

Saarialho-Kere UK, Kovacs SO, Pentland AP, Olerud JE, Welgus HG and Parks WC (1993) Cell-matrix interactions modulate interstitial collagenase expression by human keratinocytes actively involved in wound healing. *J Clin Invest* **92**: 2858–2866

Saarialho-Kere UK, Pentland AP, Birkedal-Hansen H, Parks WC and Welgus HG (1994) Distinct populations of basal keratinocytes express stromelysin-1 and stromelysin-2 in chronic wounds. *J Clin Invest* **94**: 79–88

Sato H, Takino T, Okada Y, Cao J, Shinagawa A, Yamamoto E and Seiki M (1994) A matrix metalloproteinase expressed on the surface of invasive tumour cells. *Nature* **370**: 61–65

Sirum KL and Brinckerhoff CE (1989) Cloning of the genes for human stromelysin and stromelysin 2: differential expression in rheumatoid synovial fibroblasts. *Biochemistry* **28**: 8691–8698

Skyldberg B, Salo S, Eriksson E, Aspenblad U, Moberger B, Tryggvason K and Auer G (1999) Laminin-5 as a marker of invasiveness in cervical lesions. *J Natl Cancer Inst*, **91**: 1882–1887

Sreenath T, Matrisian LM, Stetler-Stevenson W, Gattoni-Celli S and Pozzatti RO (1992) Expression of matrix metalloproteinase genes in transformed rat cell lines of high and low metastatic potential. *Cancer Res* **52**: 4942–4947

Sympson CJ, Bissell MJ and Werb Z (1995) Mammary gland tumour formation in transgenic mice overexpressing stromelysin-1. *Semin Cancer Biol* **6**: 159–163

Tamai K, Ishikawa H, Mauviel A and Uitto J (1995) Interferon-gamma coordinately upregulates matrix metalloprotease (MMP)-1 and MMP-3, but not tissue inhibitor of metalloproteases (TIMP), expression in cultured keratinocytes. *J Invest Dermatol* **104**: 384–390

Tsukifuji R, Tagawa K, Hatamochi A and Shinkai H (1999) Expression of matrix metalloproteinase-1, -2 and -3 in squamous cell carcinoma and actinic keratosis. *Br J Cancer* **80**: 1087–1091

Vaalamo M, Karjalainen-Lindsberg ML, Puolakkainen P, Kere J and Saarialho-Kere U (1998) Distinct expression profiles of stromelysin-2 (MMP-10), collagenase-3 (MMP-13), macrophage metalloelastase (MMP-12), and tissue inhibitor of metalloproteinases-3 (TIMP-3) in intestinal ulcerations. *Am J Pathol* **152**: 1005–1014

Werb Z, Vu TH, Rinkenberger JL and Coussens LM (1999) Matrix-degrading proteases and angiogenesis during development and tumor formation. *APMIS* **107**: 11–18

Windsor LJ, Grenett H, Birkedal-Hansen B, Bodden MK, Engler JA and Birkedal-Hansen H (1993) Cell type-specific regulation of SL-1 and SL-2 genes. Induction of the SL-2 gene but not the SL-1 gene by human keratinocytes in response to cytokines and phorbolesters. *Biol Chem* **268**: 17341–17347

Yancey KB (1995) Adhesion molecules II: Interactions of keratinocytes with epidermal basement membrane. *J Invest Dermatol* **104**: 1008–1014

Yoshizaki T, Sato H, Maruyama Y, Murono S, Furukawa M and Park CS and Seiki M (1997) Increased expression of membrane type 1-matrix metalloproteinase in head and neck carcinoma. *Cancer* **79**: 139–144

*© 2001 Cancer Research Campaign* 
